# Survey of Anesthesia, Sedation, and Non-sedation Practices for Children Undergoing Repetitive Cranial or Craniospinal Radiotherapy

**DOI:** 10.7759/cureus.24075

**Published:** 2022-04-12

**Authors:** Pascal Owusu-Agyemang, January Y Tsai, Ravish Kapoor, Antoinette Van Meter, Gee Mei Tan, Sarah Peters, Lucas Opitz, Dino Pedrotti, Hernando S DeSoto, Acsa M Zavala

**Affiliations:** 1 Anesthesiology and Perioperative Medicine, MD Anderson Cancer Center, Houston, USA; 2 Anesthesiology, Children's Hospital Colorado, Aurora, USA; 3 Department of Particle Therapy, Essen University Hospital, Essen, DEU; 4 Anesthesiology, Centre Antoine Lacassagne, Nice, FRA; 5 Anesthesiology, St. Chiara Hospital, Azienda Provinciale per i Servizi Sanitari (APSS), Trento, ITA; 6 Anesthesiology, The University of Florida Health Science Center Jacksonville, Jacksonville, USA

**Keywords:** radiotherapy, sedation, anesthesiology, repetitive, pediatrics

## Abstract

Background

Children undergoing cranial or craniospinal radiotherapy may require over 30 treatments within a six-week period. Facilitating these many treatments with the patient under anesthesia presents a significant challenge, and the most preferred anesthetic methods remain unknown. The primary goal of this study was to determine the most preferred anesthetic methods and agents for children undergoing daily cranial or craniospinal radiotherapy.

Methods

An 83-item web-based survey was developed. An introductory email was sent to 505 physicians and child-life specialists with expertise in pediatric anesthesia and/or affiliated with pediatric radiation oncology departments.

Results

The response rate was 128/505 (25%) and included specialists from Africa (5, 4%), Asia (18, 14%), Australia/Oceania (5, 4%), Europe (45, 35%), North America (50, 39%), and South America (5, 4%). The 128 respondents included 91 anesthesiologists (71%), 20 physicians who were not anesthesiologists (16%), 14 child life/social education specialists (11%), one radiotherapist, one pediatric radiation nurse, and one non-specified medical professional (all = 2%). Of the 128 respondents, 95 (74%) used anesthesia or sedation to facilitate repetitive cranial or craniospinal radiotherapy. Overall, total intravenous anesthesia without intubation was preferred by 67 of 95 (71%) specialists during one or more forms of radiotherapy. During photon-based radiotherapy, total intravenous anesthesia without intubation was the preferred anesthetic method with the patient in the supine (57/84, 68%) and prone positions (25/40, 63%). Propofol was the most used anesthetic agent for both supine (73/84, 87%) and prone positions (38/40, 95%). For proton radiotherapy, total intravenous anesthesia without intubation was the most preferred anesthetic method for the supine (32/42, 76%) and prone treatment positions (11/18, 61%), and propofol was the most used anesthetic (supine: 40/43, 93%; prone: 16/18, 89%).

Conclusions

In this survey of 95 specialists responsible for anesthesia or sedation of children undergoing repetitive cranial or craniospinal radiotherapy, propofol-based total intravenous anesthesia without intubation was the preferred anesthetic technique.

## Introduction

Anesthesia for children undergoing repetitive cranial or craniospinal radiotherapy presents substantial challenges [1]. In addition to the remote location of radiotherapy units and the use of conformational masks that impede access to the airway, children may have to undergo 30 or more treatments over a six-week period [2].

Preferably, anesthesia in children undergoing repetitive radiotherapy should ensure comfort and immobility, and employ a safe and replicable form of airway management [3]. These preconditions have been met in several ways and have evolved over the years. For example, the routine use of agents such as halothane [4], methoxyflurane [5], barbiturates [6], and intramuscular ketamine [7] has been replaced with the use of propofol and sevoflurane [1,8-9]. Regarding airway management, the introduction of the laryngeal mask airway has provided an alternative to daily endotracheal intubations or anesthesia with an unprotected airway [1,3].

Currently, relatively little is known about which anesthetic methods are preferred by specialists who provide anesthesia for children undergoing repetitive cranial or craniospinal radiotherapy. To the best of our knowledge, our previously published limited survey on anesthesia for proton radiotherapy is the only study that has explored anesthesia practice preferences in a similar setting [10]. In that survey, a slight majority of respondents (8/14, 57%) preferred total intravenous anesthesia (TIVA) with an unprotected airway. However, proton radiotherapy tends to be longer in duration than photon radiotherapy and is usually performed at standalone centers. Thus, practice preferences may differ for photon radiotherapy.

To that end, we expanded upon our previously published survey by including questions about photon radiotherapy and by inviting the participation of representatives from previously surveyed and newer proton radiotherapy centers. Our primary goal was to determine the preferred anesthetic methods and commonly administered anesthetics during cranial and craniospinal radiotherapy in children. Other important aspects of radiotherapy management, including non-anesthetic methods, procedure scheduling, pre-procedural assessment, fasting guidelines, staffing models, monitoring, and patient recovery, and the impact of facility infrastructure on anesthetic management were also surveyed.

## Materials and methods

Development of the survey

The institutional review board of The University of Texas MD Anderson Cancer Center issued a written determination of exemption for this survey (Institutional Review Board #2019-0927; Chairperson: Dr. Jennifer Litton, The University of Texas MD Anderson Cancer Center, 1515 Holcombe Boulevard, Houston, TX 77030, email , telephone 713-792-2517).

Using the Research Electronic Data Capture system (REDCap; Vanderbilt University, Nashville, TN), the study authors developed a web-based survey and tested it for functionality. Functionality was tested by three of the co-authors (AZ, RK, and AVM) by responding to the survey questionnaire during various stages of its development. Errors in the design of the survey instrument were noted and corrected. The total number of survey items was 83 (Appendix A). Not all questions were mandatory, and branching logic was used to explore respondent preferences and limit the number of questions that were not applicable to a particular respondent. For example, if a respondent indicated they did not personally administer sedatives or anesthetics (Appendix A, item #5), they would be directed toward questions about non-sedative methods of facilitating radiotherapy.

Two emails were composed. The first, an introductory email, was an invitation to participate in the survey or help identify an anesthesiologist and/or child life specialist who would be willing to participate in the survey (Appendix B). The second email, which contained a description of the survey, a consent statement, and a unique link to the web-based survey (Appendix C), was sent when a respondent agreed to participate in the survey. Administration of the survey adhered to the Checklist for Reporting Results of Internet E-Surveys [11].

Targeted participants

The authors agreed upon three target groups. The first group was attendees of the First International Meeting on Iterative Pediatric Anesthesia who provided email addresses for further correspondence. The second group was authors of recent (2015-2020) scientific publications on anesthetic and non-anesthetic methods of facilitating radiotherapy in children, pediatric radiation oncology, or surveys on topics in anesthesiology. The third group was pediatric anesthesiologists, pediatric radiation oncologists, and child-life or social education specialists who were practicing at member centers of the Children’s Oncology Group, the European Society for Paediatric Oncology, Particle Therapy Co-Operative Group, and the Paediatric Radiation Oncology Society. Institutional participation was limited to only one specialist responsible for anesthesia and/or another responsible for facilitating treatment without anesthesia. The survey was initially open from February 2, 2020, to May 2, 2020. However, due to the limited number of responses (presumably due to the emergence of the coronavirus disease 2019 (COVID-19) pandemic), the survey was reopened between October 3, 2021, and January 3, 2022. An automatic email reminder was sent every 14 days over the survey time periods.

Data storage, management, and analysis

No respondent identifiers were associated with the reported survey responses. The data were stored and analyzed using tools of the REDCap system. Survey responses that did not provide details of anesthetic or non-anesthetic management were considered incomplete and excluded from the analysis. Completed surveys were analyzed and presented as frequencies and percentages.

## Results

Respondent characteristics

A total of 505 introductory emails were sent. Two hundred and sixteen of the email recipients (43%) either indicated a willingness to participate in the survey or provided the contact information of a specialist who was willing to participate. The second email containing the consent statement and a link to the survey was sent to these 216 potential participants.

The total number of surveys received was 134. Of these, six provided no details of anesthetic or non-anesthetic management and were excluded from the analysis. As a result, the survey response rate was 128/505, or 25%. Respondent practice locations were Africa (5, 4%), Asia (18, 14%), Australia/Oceania (5, 4%), Europe (45, 35%), North America (50, 39%), and South America (5, 4%). The 128 respondents included 91 anesthesiologists (71%), 20 physicians who were not anesthesiologists (16%), 14 child life/social education specialists (11%), one radiotherapist, one pediatric radiation nurse, and one non-specified medical professional (all = 2%).

Of the 128 respondents, 95 (74%) used anesthesia or sedation to facilitate repetitive cranial or craniospinal radiotherapy in children; 52 at photon radiotherapy centers (55%), 10 at proton radiotherapy centers (10%), and 33 at both photon and proton radiotherapy centers (35%). The remaining respondents (33/128, 26%) used non-sedative methods to facilitate treatment; 17 at photon radiotherapy centers (52%), six at proton radiotherapy centers (18%), nine at both photon and proton radiotherapy centers (27%), and one other practice location (3%) which was not specified.

Fasting guidelines and non-pharmacologic pre-procedural anxiolysis

**Figure 1:**
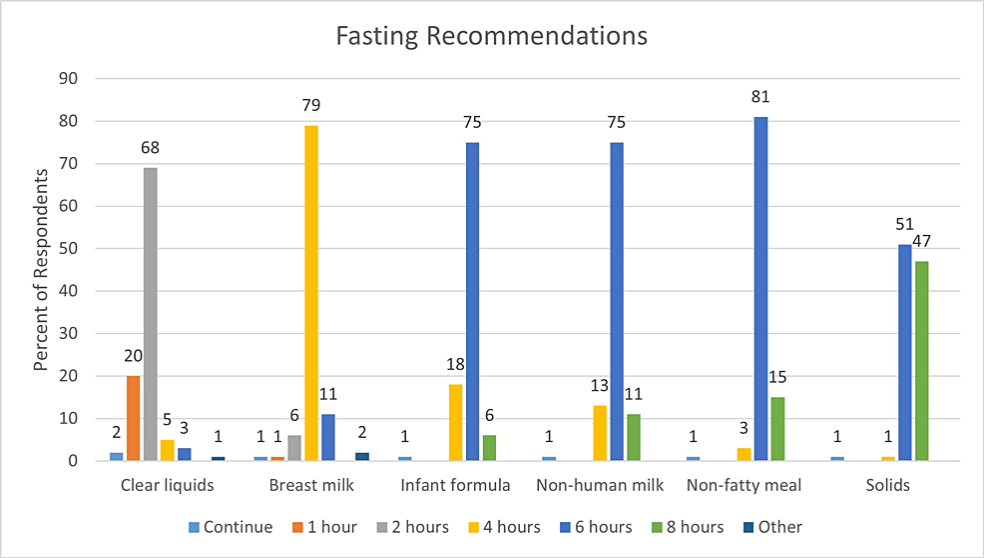
Fasting guidelines (95 respondents) The most common recommendations were two hours for clear liquids (65/95, 68%), four hours for breast milk (75/95, 79%), six hours for infant formula (71/95, 75%), six hours for non-human milk (70/93, 75%), six hours for light (non-fatty) meals (77/95, 81%), and six hours for solid foods (48/93, 52%)

The fasting recommendations of the 95 respondents who facilitated radiotherapy with anesthesia or sedation are shown in Figure 1.

The most common recommendations were two hours for clear liquids (65/95, 68%), four hours for breast milk (75/95, 79%), six hours for infant formula (71/95, 75%), six hours for non-human milk (70/93, 75%), six hours for light (non-fatty) meals (77/95, 81%), and six hours for solid foods (48/93, 52%). Fasting recommendations labeled as “other” included clear liquids up to 30 minutes before induction (1/95, 1%) and withholding of breast milk for three hours (2/95, 2%). One respondent (1%) who provided only conscious sedation did not recommend any fasting.

Fifty-one of the 95 respondents who used anesthesia to facilitate radiotherapy (54%) routinely used a combination of non-pharmacologic methods to alleviate anxiety prior to treatment. Methods included allowing family members in the treatment area (48/95, 51%), allowing the child to use an iPad or tablet (43/95, 45%), having a child-life or social education specialist present (27/95, 28%), adhering to routines familiar to the child (1/95, 1%), having a music therapist present (1/95, 1%), letting the child listen to preferred music playlists (1/95, 1%), using child-friendly language (1/95, 1%), promises of fulfilling an established reward system (2/95, 2%), and using pediatric hypnotic techniques (1/95, 1%).

Anesthetic techniques

**Figure 2:**
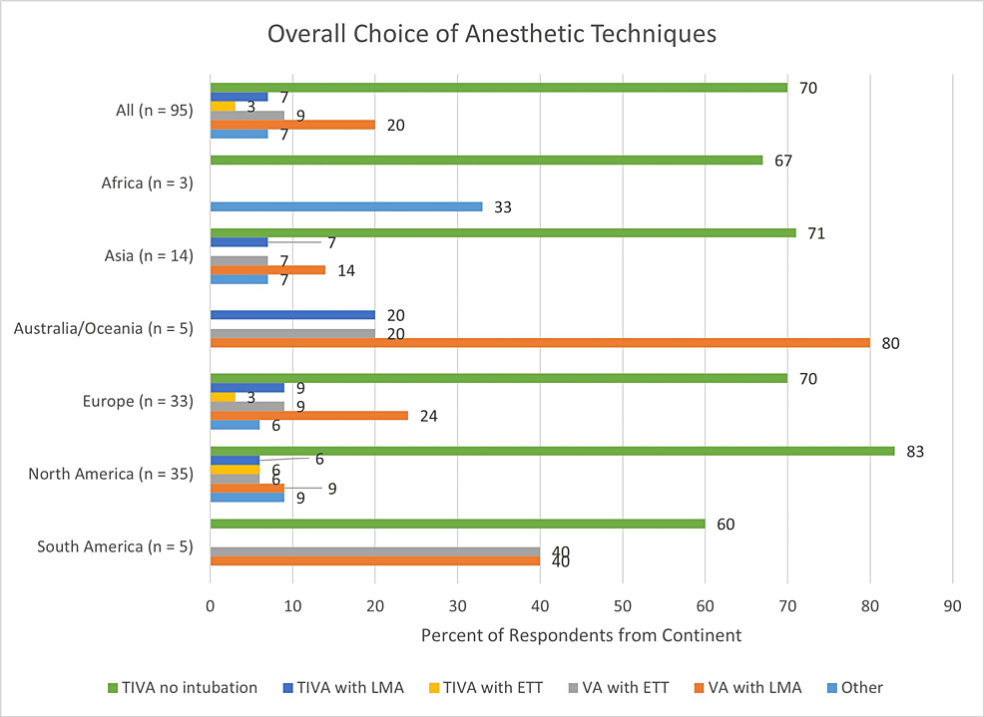
Overall choice of anesthetic techniques TIVA, total intravenous anesthesia; LMA, laryngeal mask airway; ETT, endotracheal tube; VA, volatile anesthetics

Overall, 67 of the 95 specialists (70%) who provided anesthesia or sedation preferred TIVA without intubation for one or more forms of radiotherapy. The overall frequency of choice of anesthetic techniques is shown in Figure 2.

Anesthesia for cranial and craniospinal photon-based radiotherapy

**Figure 3:**
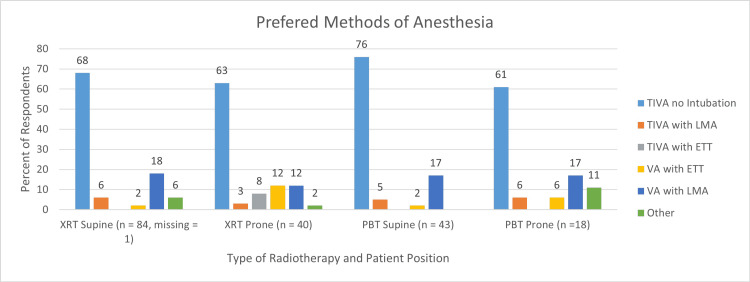
Preferred methods of anesthesia during cranial or craniospinal radiotherapy TIVA, total intravenous anesthesia; LMA, laryngeal mask airway; ETT, endotracheal tube; VA, volatile anesthetics; XRT, photon radiotherapy; PBT, proton radiotherapy; n, number of respondents TIVA without intubation was the most preferred anesthetic option.

Eighty-four of the 85 respondents (99%) who provided anesthesia for photon radiotherapy provided details of their anesthetic preferences. The majority (57/84, 68%) preferred TIVA without intubation for procedures in the supine position (Figure 3).

**Figure 4:**
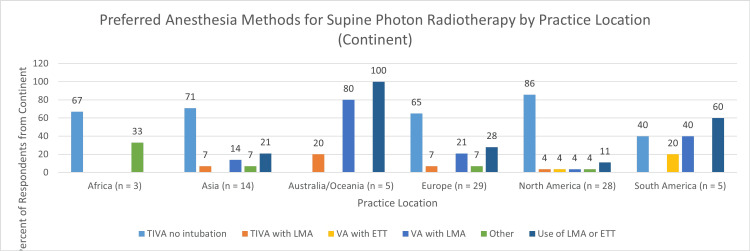
Preferred methods of anesthesia for supine photon radiotherapy by practice location TIVA, total intravenous anesthesia; LMA, laryngeal mask airway; ETT, endotracheal tube; VA, volatile anesthetics

An endotracheal tube (ETT) or laryngeal mask airway (LMA) was preferred by 22/84, or 26%. Preference for an ETT or LMA was more common outside North America; 19/56 (34%) versus 3/28 (11%), Figure 4.

Preferences described as “other” included intravenous (IV) midazolam alone (2/84, 2%), and one each (1%) of propofol with nasal cannula when intravenous access was available or LMA and sevoflurane in the absence of intravenous access, volatile anesthesia with a face mask, and a dexmedetomidine infusion with propofol boluses.

Propofol was the most routinely administered anesthetic for treatments in the supine position (73/84, 87%). The use of other anesthetic agents and drugs is illustrated in Table 1.

**Table 1:** Commonly administered anesthetics during photon radiotherapy in the supine position Data expressed as % of respondents from the continent. IV: intravenous

	Commonly Administered Anesthetics during Photon Radiotherapy Supine Position						
Drug	All (%) (n = 84)	Africa (%) (n = 3)	Asia (%) (n = 14)	Australia/Oceania (%) (n = 5)	Europe (%) (n = 29)	N. America (%) (n = 29)	S. America (%) (n = 5)
Propofol	87	100	77	80	90	93	60
Dexmedetomidine	20	33	21		21	25	
Opioids	8		7		3	18	
Anticholinergics	11	33	14		10	7	20
IV Midazolam	26	67	57		17	21	20
Oral Midazolam	7		7		10	7	
Ketamine	17	33	36		14	11	20
Barbiturates	1		7				
Halothane	1						20
Sevoflurane	27		21	80	35	14	40
Isoflurane	1						20
Prophylactic Antiemetics	26	33	21	40	21	32	20
Other	2		7			4	

Seven of the 85 respondents who provided anesthesia for photon-based radiotherapy in the supine position were not anesthesiologists. They included three radiation oncologists practicing in Asia (2/7, 29%), one radiation oncologist in Europe (1/7, 14%), and four other physicians practicing in Asia (1/7, 14%), Europe (1/7, 14%), North America (1/7, 14%), and South America (1/7, 14%) whose specialties were not specified. Their preferred methods of anesthesia/sedation were TIVA without intubation (2/7, 29%), TIVA with LMA (1/7, 14%), and volatile anesthetics with an LMA (2/7, 29%), and other (2/7, 29%).

Similar to treatments in the supine position, the majority of respondents (25/40, 63%) preferred TIVA without intubation for procedures in the prone position (Figure 3). One preference (“other”) was described as TIVA with a low threshold to use an advanced airway. Of the 40 respondents who provided anesthesia for photon-based radiotherapy in the prone position, only one was not an anesthesiologist. Their preferred method of anesthesia was TIVA without intubation.

Propofol was the most routinely used anesthetic during treatments in the prone position (38/40, 95%). Other less routinely used anesthetics included sevoflurane (13/40, 33%), dexmedetomidine (10/40, 25%), intravenous midazolam (9/40, 23%), opioids (5/40, 13%), ketamine (6/40, 15%), and barbiturates (1/40, 3%). Anticholinergics were administered by five respondents (13%) and antiemetics by 11 respondents (28%).

Anesthesia for cranial or craniospinal proton radiotherapy

Forty-two of the 43 respondents who provided anesthesia for proton radiotherapy provided details of their anesthetic preferences. All 43 provided details about their drug preferences. TIVA without intubation was the most preferred anesthetic option for procedures administered with the patient in the supine (32/42, 76%) or prone (11/18, 61%) positions (Figure 3). “Other” preferences for procedures in the prone position were TIVA with a natural airway and low threshold to use an advanced airway, and TIVA without intubation or intubation with volatile anesthetics (all 2/18 or 11%).

Anesthetics and drugs routinely used for procedures in the supine position are shown in Table 2.

**Table 2:** Commonly administered anesthetics during proton radiotherapy in the supine position Data expressed as % of respondents from the continent. IV: intravenous

	Commonly Administered Anesthetics during Proton Radiotherapy Supine Position			
Drug	All (%) (n = 43)	Asia (%) (n = 3)	Europe (%) (n = 16)	N. America (%) (n = 24)
Propofol	93	67	88	100
Dexmedetomidine	26		25	29
Opioids	7		13	4
Anticholinergics	9		6	13
IV Midazolam	28	67	38	17
Oral Midazolam	9		19	4
Ketamine	7	33	6	4
Barbiturates	5	33	6	
Sevoflurane	28	33	50	13
Isoflurane				
Prophylactic Antiemetics	33		31	38
Other	7	33	6	4

For procedures in the prone position, propofol was the most routinely used agent (16/18, 89%), followed by intravenous midazolam (8/18, 44%), sevoflurane (7/18, 39%), dexmedetomidine (6/18, 33%), opioids (3/18, 17%), ketamine (1/18, 6%), anticholinergics (2/18, 11%), and antiemetics (4/18, 22%).

Of the 43 respondents who provided anesthesia at proton radiotherapy centers, only two (5%) were not anesthesiologists. They included a radiation oncologist practicing in Asia and a physician in North America whose specialty was not specified. Both preferred TIVA without intubation for procedures in the supine position. None administered anesthesia for procedures in the prone position.

Monitoring

**Figure 5:**
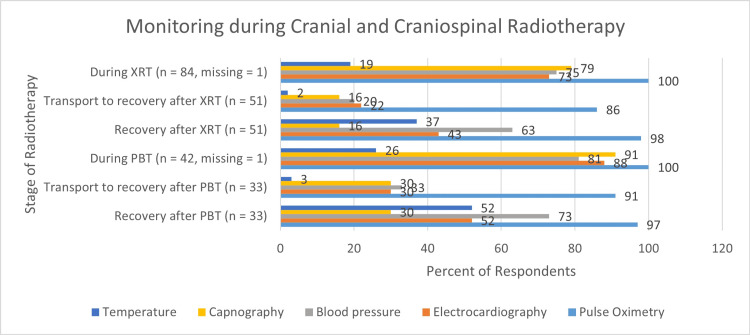
Monitoring during Cranial and Craniospinal Radiotherapy XRT, photon radiotherapy; PBT, proton radiotherapy; n, number of respondents. Pulse oximetry was used by all respondents during treatments.

Pulse oximetry was used by all respondents during treatment sessions. Compared with other monitors, pulse oximetry had the highest use during all phases of care (Figure 5).

**Figure 6:**
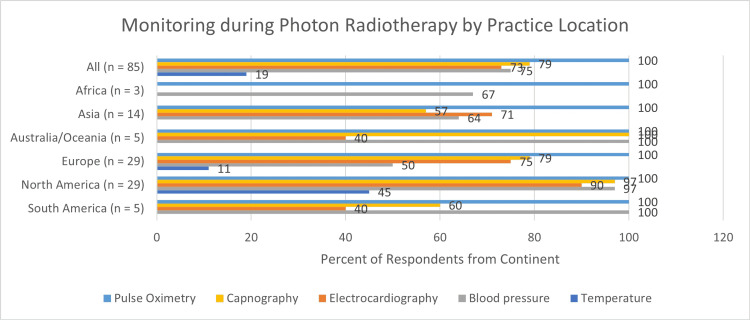
Monitoring during photon radiotherapy by practice location

During treatment sessions, the most common combination of monitoring was capnography, pulse oximetry, blood pressure monitoring, and electrocardiography. This combination of monitors was used by 51 of 85 respondents (60%) during photon radiotherapy and 32 of 43 respondents (74%) during proton radiotherapy. All standard monitors including temperature were used by 16 of 85 respondents (19%) during photon radiotherapy and 11 of 43 respondents (26%) during proton radiotherapy. The use of monitors by practice location (continents) is shown in Figure 6.

Few respondents had the ability to remotely control the functions of their anesthesia monitors from outside the treatment gantry; 29 of 84 respondents (35%) at photon radiotherapy facilities, and 19 of 42 respondents (45%) at proton radiotherapy facilities.

Non-sedation methods of facilitating radiotherapy

Thirty-three respondents (26%) facilitated repeated radiotherapy sessions without anesthesia or sedatives. They included 14 child-life/social education specialists (42%), three anesthesiologists (9%), 14 physicians who were not anesthesiologists (42%), one pediatric radiotherapist (3%), and one pediatric radiation oncology nurse (3%). Non-sedation methods were used in Africa (2/33, 6%), Asia (4/33, 12%), Europe (12/33, 36%), and North America (15/33, 46%). Expressed as a proportion of survey participants from a particular continent, this represented 2/5 or 40% from Africa, 4/18 or 22% from Asia, 12/45 or 27% from Europe, and 15/50 or 30% from North America.

An equal number of respondents were comfortable attempting non-sedation above the ages of three, five, and six years (each 7/33, or 21%). Nine respondents (27%) preferred four years as a minimum age, one respondent (3%) preferred eight years, while another two respondents (6%) used a combination of factors to decide the appropriateness of treatments without sedation, not just age.

Pre-treatment counseling was used by 29 of the 33 respondents (88%) who facilitated treatment without sedation. Other methods of preparation included visits to the treatment room (29/33, 88%) and observing other children undergoing radiotherapy (9/33, 27%). Successful completion of treatment was carried out with the aid of storybooks (15/33, 46%), video games (8/33, 24%), movies (14/33, 42%), hypnotherapy (1/33, 3%), and audio/visual interaction with parents (21/33, 64%). Additional details about methods of achieving successful radiotherapy without anesthesia or sedation are described in Table 3.

**Table 3:** Methods for preparation and completion of radiotherapy without sedation in children undergoing repetitive cranial or craniospinal radiotherapy.

Preparation for radiotherapy	During radiotherapy
Immobilization devices, practice mask on a doll or action figure, parents present, pictures, videos	Music/customized playlists
Show children pictures of the machine and medical materials (such as the radiation mask). For a child on the cusp of needing anesthesia, have the child practice lying still in an empty treatment room	Because patients are unable to have any visual distraction owing to the placement and movement of the machine, children listen to music or audiobooks as a form of distraction. Child-life specialists coach children throughout their treatments (verbal encouragement, giving them information on what to expect step by step). Caregivers/parents are not allowed to stay in the treatment room once the child is in treatment position and ready to start
	Music, Podcasts, Audiobooks, Breathing techniques
Medical play and treatment practice sessions inside the computed tomography room leading up to computed tomography simulation, and inside the treatment room leading up to the start of treatment. Creation of a practice mask for patients to take home	Music/audio recordings, small fidget items (stress balls, fidget cubes, etc.), or comfort items. Anticipatory guidance/narration during treatment by staff. Most importantly, an individualized coping plan that the child actively participates in creating each day but especially as the treatment is starting
	Focused more on audio diversion than video games or movies. Playlists, audiobooks, podcasts, recordings of parents talking or reading, listening to movies or YouTube videos
	Because the child must remain still to receive proton radiotherapy, it is very difficult to use typical distraction items such as books, movies, or toys. Adequate preparation, including developmentally appropriate verbal explanation, photos, medical play, and/or rehearsal of coping techniques, are all key components in ensuring successful radiotherapy without anesthesia/sedation. In addition to this preparation, a child-life specialist must be present in the treatment room alongside radiation therapists to offer step-by-step instructions, explanations, encouragement, and positive verbal praise in a manner that is child-friendly and developmentally appropriate to ensure cooperation, positive coping, and mastery of the experience
	A registered health play specialist makes an individualized play/support plan as part of the preparation and this includes music, podcasts, or audiobooks; breathing or relaxation techniques; visualization; and play to demystify the environment. Daily health play specialist support is used throughout treatment, which is then handed over to treatment radiographers

Scheduling of daily radiotherapy sessions with anesthesia

At most institutions, scheduling pediatric radiotherapy treatments with anesthesia was the responsibility of the anesthesiologists or physicians responsible for sedation (51/95, 54%). Radiation oncologists (17/95, 18%) and radiotherapists (19/95, 20%) were also responsible but less often. Other scheduling responsibilities involved coordination between the departments of anesthesiology and radiation oncology (10/95, 11%), and in one case, this was facilitated with the help of a nurse liaison.

Ninety-four respondents provided information about how late in the day radiotherapy was scheduled to start. Among these 94 respondents, 83 (88%) indicated a specific cutoff time. The remaining 11 respondents (12%) indicated no specific cutoff times. Where enforced, cutoff times were as follows: first one to two patients of the day (2/94, 2%), between 8 and 10 am (1/94, 1%), not after 10 am (1/94, 1%), not after 12 pm (40/94, 43%), not after 1 pm (1/94, 1%), between 12 pm and 2 pm (1/94, 1%), not after 3 pm (19/94, 20%), and not after 5 pm (18/94, 19%).

Pre-procedural assessment

Less than half of the physicians responsible for anesthesia or sedation (36/94, 38%) routinely participated in interdisciplinary meetings to discuss patient care issues, and psychoeducational counseling was offered to just over half of children and families (57/93, 61%).

Answers to questions about pre-procedural testing were provided by 79 of the 95 physicians (83%) who facilitated treatment with anesthesia or sedation. One or more of the following tests was required before the first anesthetic was administered: complete blood count (59/79, 75%), serum electrolytes (42/79, 53%), serum albumin (12/79, 15%), and chest x-ray (17/79, 22%). Ten respondents (13%) did not routinely require any pre-procedural testing.

Other tests included screening for multidrug-resistant pathogens and creatinine clearance (1/79, 1%), pregnancy tests in female patients aged 10 years or older (1/79, 1%), and cardiac ultrasound (1/79, 1%). A clinical examination and medical clearance by a pediatrician were required by two respondents (2%). Eight respondents (10%) indicated that their decision to require extra testing was dependent on the patient's condition.

Staffing at radiotherapy facilities

Anesthesia/sedation teams at photon radiotherapy facilities were composed of one to six members, with two members being most common (43/84, 51%). At four institutions (5%), a physician was the sole member of the anesthesia or sedation team. At a limited number of locations (5/84, 6%), the sedation team included an anesthesiologist as well as a physician who was not an anesthesiologist. This combination of staffing was represented in Europe (2/29, 7%), North America (1/29, 3%), and South America (2/5, 40%). Nurses dedicated to the recovery room were present at 44/84 or 52% of facilities, and a designated emergency medical response team had been formed within 18/29 or 62% of the facilities (missing = 56).

Of the 43 respondents who practiced at proton radiotherapy centers, 42 (98%) provided details about their anesthesia/sedation teams. Anesthesia/sedation teams included one or more of the following: anesthesiologists (40/42, 95%), physicians who were not anesthesiologists (4/42, 9%), certified registered nurse anesthetists (CRNAs) (29/42, 69%), anesthesia assistants (4/42, 9%), anesthesia residents (7/42, 17%), registered nurses (17/42, 41%), anesthesia technologists (6/42, 14%), recovery room nurses (32/42, 76%), designated emergency medical response team within the facility (11/23 or 48%, missing = 20), and other unspecified staffing models (2/42, 5%). The number of team members on a given day ranged from one to six, with two and three being the most common (each 15/42, or 36%).

Photon radiotherapy facilities

Most photon radiotherapy facilities are within a main hospital (54/84, 64%). Infrastructure and resources at treatment locations included: separate induction rooms (14/84, 17%), treatment room oxygen connected to central oxygen supply (71/84, 85%), wall suction outlets in treatment rooms (70/84, 83%), a ventilator in the treatment room (56/84, 67%), anesthetic gas scavenging (40/84, 48%), a dedicated recovery room (51/84, 61%), advanced pediatric airway equipment (59/84, 70%), defibrillator with pediatric pads/paddles (55/84, 66%), dedicated pediatric cardiopulmonary resuscitation cart (46/84, 55%), and malignant hyperthermia cart (23/84, 27%).

Transportation from the treatment area to the recovery room ranged from less than a minute to 15 minutes. Of the 51 facilities with dedicated recovery rooms, all were equipped with a central oxygen supply, 96% with wall suction outlets, and 98% with monitors. Additional details of photon radiotherapy facility infrastructure and equipment are shown in Table 4.

**Table 4:** Photon radiotherapy facility infrastructure and equipment Data expressed as a percentage of respondents practicing in the continent

Facility Infrastructure and Equipment	Practice location						
	All (n = 85)	Africa (n = 3)	Asia (n = 14)	Australia/Oceania (n = 5)	Europe (n = 29)	North America (n = 29)	South America (n = 5)
Separate induction room	17		14		21	18	20
Treatment room oxygen connected to central oxygen supply	85	67	71	80	93	86	80
Wall suction outlet in the treatment room	83	67	86	100	79	86	80
Ventilator in the treatment room	67	33	36	100	72	75	60
Anesthetic gas scavenging in the treatment room	48	33	14	80	48	57	60
Dedicated recovery room	61	67	50	80	59	61	80
Recovery room nurse	86	100	86	100	82	88	75
Recovery room central oxygen	100	100	100	100	100	100	100
Recovery room wall suction	96	100	100	100	94	94	100
Recovery room monitors	98	100	100	75	100	100	100
Advanced pediatric airway equipment	70	67	57	80	79	64	80
Defibrillator with pediatric pads/paddles	66	33	50	100	62	79	40
Dedicated pediatric cardiopulmonary resuscitation cart	55		29	60	59	68	60
Malignant hyperthermia cart	23		14	40	17	43	40

Proton radiotherapy facilities

Most proton radiotherapy facilities were free-standing (24/43, 56%). Descriptions of available resources were provided by 42 of the 43 respondents and included separate induction rooms (19/42, 45%), treatment room oxygen connected to the central oxygen supply (37/42, 88%), wall suction outlets in treatment rooms (38/42, 90%), ventilator in the treatment room (31/42, 74%), anesthetic gas scavenging (28/42, 67%), dedicated recovery room (34/42, 81%), advanced pediatric airway equipment (34/42, 81%), defibrillator with pediatric pads/paddles (36/42, 86%), dedicated pediatric cardiopulmonary resuscitation cart (32/42, 76%), and malignant hyperthermia cart (24/42, 57%).

Duration of patient transport to recovery rooms ranged from less than a minute to 10 minutes. In most cases (26/34, 76%), it took three minutes or less. All recovery rooms were equipped with a central oxygen supply and monitors (100%) and wall suction outlets were available in 94% of recovery rooms.

The design or layout of the proton radiotherapy facilities negatively impacted the anesthetic practice of 17 respondents (40%). Where provided, additional details of the negative impacts are shown in Table 5.

**Table 5:** Infrastructure-related factors that affected anesthetic management during proton radiotherapy for children undergoing repetitive cranial or craniospinal radiotherapy

Factors
No evacuation system for anesthetic gases. No induction rooms
Some rooms do not have electrical outlets (sealed from the outside). Some rooms are not designed to visualize anesthesia monitors in a convenient way
No gas scavenging system. No wall suction unit. Some doors do not have automatic opening
Only one treatment gantry allows for the use of volatile anesthetics; in other treatment rooms, total intravenous anesthesia with a face mask or nasal cannula is required
Distance to the anesthetized child. Slow opening of the radiation door
Limited post-anesthesia care unit space
Frequent performance interference with monitors and medication infusion pumps as they are more often exposed to the neutron scatter during proton therapy (currently looking to replace monitors with machines capable of shielding for proton therapy)
Lack of privacy for the patient when they are leaving the gantry to return to the post-anesthesia care unit
By miscommunication, the central oxygen and air supply was not connected to the treatment rooms, even though this was discussed with the architect/builder (once the problem was discovered, it was too late/too expensive to change)
Located in the basement of the adult hospital, across the highway from the children’s hospital. Phones often do not work; unable to call for help. Equipment must be dragged across the adult medical center each morning and the setup is extensive because they are not allowed to keep any pediatric equipment in the basement
No malignant hyperthermia kit No volatile anesthetics
Limited workspace
Only 3 bays in the recovery room. The computed tomography room is too small for a stretcher. Computed tomography/magnetic resonance imaging is located on a separate floor from the treatment room/recovery room

## Discussion

In this survey, with the exception of respondents practicing in Australia/Oceania, TIVA without intubation was the most preferred anesthetic technique for children undergoing repetitive cranial or craniospinal radiotherapy. Propofol was the most commonly used anesthetic overall and across all continents. For procedures performed without anesthesia, pre-treatment counseling was used by most respondents, and treatment was accomplished with the aid of storybooks, video games, movies, hypnotherapy, and audio/visual interaction with parents and staff.

Several single-institution reports have demonstrated the safety of propofol-based TIVA with a natural airway in children undergoing repetitive radiotherapy. In one of the earliest publications on this technique, Buehrer et. al. described the safety and efficacy of a fixed rate propofol infusion in 18 children who underwent proton radiotherapy. In all treatments, supplemental oxygen was delivered with nasal prongs, and no increase in propofol requirements was observed [12]. More recently, in a large retrospective review from the Mayo Clinic in Arizona, USA, most of the 997 anesthetic procedures for radiotherapy were safely performed with single-agent propofol with an unprotected airway [9]. In another recent publication from The National Cancer Center in Gyeonggi-do, Republic of Korea, the authors described their use of targeted control infusions of propofol in 54 spontaneously breathing children undergoing a combined 1296 proton radiotherapy sessions [13]. Only six cases of transient desaturation were reported. It is, therefore, not surprising that the majority of respondents in this survey preferred this anesthetic technique.

Nonetheless, the current survey shows that the daily use of volatile anesthetics or invasive airway management for repetitive radiotherapy is also common. For example, during photon-radiotherapy in the supine position, all respondents from Australia/Oceania preferred the use of an LMA and most from South America preferred the use of an ETT or LMA. Furthermore, over 20% of respondents from Asia and Europe preferred a secure airway. This may be due to several reasons. First, this form of anesthetic management has a demonstrated track record of safety and is used as a backup method even by those who prefer TIVA with an unprotected airway [9,13]. This record of safety may be particularly important where the anesthesia is being administered at a remote location with the patient removed from the immediate reach of the primary provider. Second, the introduction of the laryngeal mask airway has provided a safe and less invasive method of airway management that avoids the morbidity associated with daily endotracheal intubations [14-15].

All respondents monitored their patients during treatment, and most continued to monitor their patients during transportation to and within recovery areas. The use of pulse oximetry during treatment sessions was universal. However, it was interesting to note that the use of capnography and electrocardiography was not universal during treatment or in the recovery areas. The clinical significance of this, if any, is unclear. However, the importance of monitoring during pediatric radiotherapy was recently highlighted by the results of the Wake Up Safe initiative [16-17]. In this study, 3379 significant adverse occurred during approximately 3.3 million anesthetics for pediatric radiotherapy. Five percent of these adverse events occurred during transportation to the recovery room, further highlighting the importance of continued monitoring over the entire peri-procedural period. It was also notable that the use of capnography was lowest in Africa, Asia, and South America. It may be reasonable to assume that this disparity is due to a lack of resources.

Regardless of the anesthesia preference, concerns about the risks associated with repetitive anesthesia and increased health care costs remain [18]. Some of the responses to this survey suggest that some children as young as three years of age may be able to undergo radiotherapy without anesthesia. Many of the methods used by our survey respondents, including storybooks, doll-size models, visits to the treatment machine, and listening to music or audiobooks, were described in a recent study that assessed the feasibility of performing radiotherapy in children without sedation [19]. In that study, the authors showed that with age-appropriate preparation, children as young as three years of age could complete radiotherapy sessions without sedation. These findings along with the results of other studies provide encouraging evidence that some younger children may be able to complete radiotherapy without anesthesia [20]. This may be particularly useful in areas of the world where access to anesthesiologists is limited. It was interesting to note that, although very limited in number, non-sedation methods were used by proportionally more respondents from Africa.

Perhaps more challenging is the ability to predict patient compliance during radiotherapy without anesthesia. To this end, Chiesa et al. described the accuracy of a multidimensional assessment tool in identifying which children were more likely to be unable to complete radiotherapy without sedation [21]. The degree of collaboration and distress noted during the medical assessment, as well as the behavioral and emotional reactions of the child upon first entering the treatment room, were most predictive of the need for anesthesia. Pre-therapy psychoeducational interventions, including pediatric hypnosis, may reduce the need for anesthesia and help children and families cope with the psychological effects of daily radiotherapy [22-23]. In the current survey, only a slight majority of centers routinely offered such support. This may be an area in need of improvement.

In the current survey, while the majority of respondents required scheduling ‘cut-off’ times in an effort to reduce the duration of fasting, less than half of these ‘cut-off’ times were during the morning hours. This suggests other scheduling constraints played a significant role in the timing of radiotherapy. With only a minority of centers having scheduling cutoff times in the morning, the effect of fasting guidelines on this patient population becomes significant [24]. Fasting recommendations of the survey respondents were mostly in accordance with current American and European guidelines. However, some authors, albeit in limited studies, have demonstrated the safety of liberal intake of clear liquids until the procedure time and allowing milk-based foods up to four hours or solid foods up to six hours before the procedure [25]. This may be an area in need of further research, especially given that staffing resources and treatment room availability continue to limit the number of children who could be treated in the morning hours.

The importance of a multidisciplinary approach to the success and safety of pediatric radiotherapy is well-documented [21]. However, as the current survey demonstrates, anesthesiologists are often not invited to discuss patient care concerns, and radiotherapy facility infrastructure-related obstacles to anesthetic management persist (Tables 4-5). Safety may be considerably improved by bridging this gap in collaboration.

The current survey has some limitations. Most importantly, it is difficult to estimate the extent to which our findings are representative of practice patterns across the globe. This is mainly due to the lack of reliable data on the number of radiotherapy centers that treat children. For example, the current membership directory of the Paediatric Radiation Oncology Society (PROS) (https://intpros.org/members/directory/) has 201 members from 54 countries, but institutional affiliation is not listed. The website of the Children’s Oncology Group lists 242 member locations across North America, Australia, New Zealand, and Saudi Arabia (https://childrensoncologygroup.org/locations), but it is not clear how many of these member locations actually provide pediatric radiotherapy. A perhaps more accurate estimate for centers in Europe was provided by a recent collaborative project across the European Society of Paediatric Oncology-affiliated countries. This project identified over 250 pediatric radiation oncology centers in Europe alone [26]. However, the centers were not listed. Estimates from Asia and the rest of the world are more difficult to find. Collaborative and quality improvement studies in this subspecialty could benefit from a more comprehensive database of centers where children are treated.

Other weaknesses of the survey include the possibility of survey bias and a low response rate, which resulted in limited representation within continents and professional groups. There are also some weaknesses in the survey design. For example, the low response rate to certain questions suggests some of the survey questions may have been difficult to understand due to language barriers or differences in the definition of professional titles.

## Conclusions

Repetitive craniospinal radiotherapy at remote locations presents unique challenges for the anesthesiologist. The results of this survey showed that propofol-based TIVA with an unprotected airway was the most preferred anesthetic technique among 95 physicians practicing at 95 different institutions for children undergoing such procedures. This survey also added to the literature showing that some children as young as three years of age may be considered for radiotherapy without sedation, thereby eliminating the risks associated with receiving multiple anesthetics over a short duration of time.

## Appendices

Appendix A: Survey

1. Participant ID text

2. Where is your radiotherapy facility located?

a.       Africa

b.       Asia

c.       Australia/Oceania

d.       Europe

e.       North America

f.        South America

3. Please indicate your specialty.  

a.       Anesthesiologist

b.       Physician non-Anesthesiologist

c.       Child Life/Social Education Specialist

d.       Other

e.       Please specify

5. Do you personally administer any sedatives or anesthetics to children undergoing repetitive cranial or craniospinal radiotherapy?

a.       Yes

b.       No

6. During which of the following therapies do you help facilitate treatment without anesthesia/sedation? Please check all that may apply

a.       Photon Radiotherapy

b.       Proton Radiotherapy

7. Above which age would you suggest an attempt at radiotherapy without anesthesia/sedation?

a.       6 years

b.       5 years

c.       4 years

d.       3 years

e.       Other

8. Please specify age at which you would attempt treatment without anesthesia/sedation.

9. Which of the following processes do you use in preparing children to undergo repetitive radiotherapy without anesthesia/sedation? Please select all that may apply.

a.       Pre-treatment counselling (by psychologist, child-life, social education, or other specialists)

b.       Visit to treatment room

c.       Observing other children undergoing radiotherapy

d.       Other

10. Please describe other methods of preparation for radiotherapy without anesthesia/sedation.

11. How do you ensure successful completion of daily treatments in children without anesthesia/sedation? Please select all that may apply.

a.       Story books

b.       Video games (Ipad/tablets)

c.       Movies

d.       Hypnotherapy

e.       Interaction with parents during treatment (audio/visual)

f.        Other

12. Please describe other means of ensuring successful radiotherapy without anesthesia/sedation.

13. Please indicate the main reason why anesthesia/sedation is not provided to the group of children that you care for?

a.       Lack of specialists who can provide sedation to children

b.       Insurance does not cover sedation/anesthesia services for radiotherapy

c.       Other

14. Please describe.

15. Thank you very much for completing this survey. Please check 'Yes' if you have provided all of your answers and wish to submit the survey. Otherwise, kindly return to any of the preceding questions to answer any that you wish to respond to.

1 Yes

Stop actions on 1

16. During which of these treatments do you administer anesthesia or sedation?

a.       Photon radiotherapy

b.       Proton radiotherapy

c.       Both proton and photon radiotherapy

17. At your institution, who has the leadership role in creating the daily anesthesia/sedation schedule for radiotherapy?

a.       Anesthesiologists/physicians responsible for sedation

b.       Radiation oncologists

c.       Radiotherapists

d.       Other

18. Please specify who is responsible for creating the daily anesthesia/sedation schedule for radiotherapy.

19. How late in the day is the last pediatric radiotherapy case scheduled?

a.       We schedule children regardless of how late in the day

b.       Not after 12 pm

c.       Not after 3 pm

d.       Not after 5 pm

e.       Other

20. Please specify how late in the day the last pediatric radiotherapy case is scheduled.

21. Is your anesthesia/sedation team invited to take part in interdisciplinary meetings to discuss patient care issues such as treatment planning, anticipated side effects of treatment, etc?

a.       Yes

b.       No

22. Is pre-treatment psychoeducational counselling routinely offered to children and/or their families?

a.       Yes

b.       No

23. Which of the following tests would you require before delivery of the first anesthetic. Please select all that may apply.

a.       Complete blood count

b.       Serum electrolytes

c.       Serum albumin

d.       Chest x-ray

e.       Other

24. Please specify which other tests you would require prior to the first day of anesthesia/sedation.

25 Section Header: Please describe your fasting guidelines

Clear liquids

a.       We do not encourage stopping

b.       1 hour

c.       2 hours

d.       4 hours

e.       6 hours

f.        8 hours

g.       Other

26. Breast milk

a.       We do not encourage stopping

b.       1 hour

c.       2 hours

d.       4 hours

e.       6 hours

f.        8 hours

g.       Other

27. Infant formula

a.       We do not encourage stopping

b.       1 hour

c.       2 hours

d.       4 hours

e.       6 hours

f.        8 hours

g.       Other

28. Non-human milk

a.       We do not encourage stopping

b.       1 hour

c.       2 hours

d.       4 hours

e.       6 hours

f.        8 hours

g.       Other

29. Light (non-fatty) meal

a.       We do not encourage stopping

b.       1 hour

c.       2 hours

d.       4 hours

e.       6 hours

f.        8 hours

g.       Other

30. Solids

a.       We do not encourage stopping

b.       1 hour

c.       2 hours

d.       4 hours

e.       6 hours

f.        8 hours

g.       Other

31. Please specify your fasting guidelines.

32. Section Header: Photon Radiotherapy Facility Design, Staffing, and Equipment

Is your photon radiotherapy facility free-standing?

a.       Yes

b.       No

33. Which of the following are present at your photon radiotherapy location? Please select all that may apply.

a.       Separate induction room

b.       Treatment room oxygen connected to central oxygen supply

c.       Wall suction outlet in treatment room

d.       Ventilator in treatment room

e.       Anesthetic gas scavenging in treatment room

f.        Dedicated recovery room

g.       Advanced pediatric airway equipment

h.       Defibrillator with pediatric pads/paddles

i.         Dedicated pediatric cardiopulmonary resuscitation cart

j.         Malignant hyperthermia cart

34. Which of the following personnel are on your anesthesia/sedation team during photon radiotherapy? Please select all that may apply.

a.       Anesthesiologist

b.       Physician non-anesthesiologist

c.       Certified Nurse Anesthetist

d.       Anesthesia Assistant

e.       Anesthesiology Resident

f.        Registered

g.       Nurse/Nurses

h.       Anesthesia Technologist

i.         Other

35. Please specify the other personnel on your anesthesia/sedation team during photon radiotherapy.

36. Is there a designated emergency medical response team within the facility where you provide anesthesia/sedation for photon radiotherapy facility?

37. Section Header: Anesthesia, Sedation, and Monitoring during Photon Radiotherapy

Do you routinely utilize non-pharmacologic methods to alleviate child anxiety before induction of anesthesia?

a.       Yes

b.       No

38. Which of the following non-pharmacologic methods do you routinely use to alleviate child anxiety before photon radiotherapy? Please select all that may apply

a.       Allowing family members into the treatment area

b.       The use of iPads/tablets/videos

c.       Child life/social education specialists

d.       Other

39. Please specify other non-pharmacologic methods of alleviating child anxiety prior to the start of photon radiotherapy.

40. What is your preferred method of anesthesia/sedation for children undergoing photon radiotherapy in the supine position?

a.       Total intravenous anesthesia without intubation

b.       Total intravenous anesthesia with Laryngeal Mask Airway

c.       Total intravenous anesthesia with endotracheal intubation

d.       Volatile anesthetics with endotracheal intubation

e.       Volatile anesthetics with Laryngeal Mask Airway

f.        Other

41. Please specify your preferred method of anesthesia for photon radiotherapy in the supine position.

42. Which of the following agents do you routinely administer during photon radiotherapy in the supine position? Please select all that may apply.

a.       Propofol

b.       Dexmedetomidine

c.       Opioids

d.       Anticholinergics

e.       IV Midazolam

f.        Oral midazolam

g.       Ketamine

h.       Barbiturates

i.        Halothane

j.        Sevoflurane

k.       Isoflurane

l.        Deflurane

m.     Antiemetics

n.       Other

43. Please specify other preferred drug notes

44. Do you administer anesthesia/sedation for children undergoing

photon radiotherapy in the prone position?

a.       Yes

b.       No

45. What is your preferred method of anesthesia/sedation for children undergoing photon radiotherapy in the prone position?

a.       Total intravenous anesthesia without intubation

b.       Total intravenous anesthesia with Laryngeal Mask Airway

c.       Total intravenous anesthesia with endotracheal intubation

d.       Volatile anesthetics with endotracheal intubation

e.       Volatile anesthetics with Laryngeal Mask Airway

f.        Other

46. Please specify your preferred method of anesthesia for photon radiotherapy in the prone position.

47. Which of the following agents do you routinely administer during photon radiotherapy in the prone position? Please select all that may apply.

a.       Propofol

b.       Dexmedetomidine

c.       Opioids

d.       Anticholinergics

e.       IV Midazolam

f.        Oral midazolam

g.       Ketamine

h.       Barbiturates

i.        Halothane

j.        Sevoflurane

k.       Isoflurane

l.        Desflurane

m.      Antiemetics

n.       Other

48. Please specify other preferred drug

49. Approximately how many minutes does it take to transport a child from the treatment gantry to the dedicated recovery room after photon radiotherapy?

50. Which of the following are available at the dedicated recovery room after photon radiotherapy under anesthesia/sedation? Please select all that may apply.

a.       Dedicated recovery room nurse

b.       Central oxygen supply

c.       Wall suction

d.       Monitors

51. Section Header: Which of the following monitors do you use? Please select all that may apply.

a.       During photon radiotherapy

b.       Pulse Oximetry

c.       ECG

d.       Blood pressure

e.       Capnography

f.        Temperature

g.       None

52. During transport to the recovery room after photon radiotherapy

a.       Pulse Oximetry

b.       ECG

c.       Blood pressure

d.       Capnography

e.       Temperature

f.        None

53. In the recovery room after photon radiotherapy?

a.       Pulse Oximetry

b.       ECG

c.       Blood pressure

d.       Capnography

e.       Temperature

f.        None

54. During photon radiotherapy, are you able to control the functions of your anesthesia monitor from outside the treatment gantry?

a.       Yes

b.       No

55. Section Header: Proton Radiotherapy Facility Design, Staffing, and Equipment

Is your proton radiotherapy facility free-standing?

a.       Yes

b.       No

56. Which of the following are available at your proton radiotherapy facility/location? Please select all that may apply.

a.       Separate induction room

b.       Treatment room oxygen connected to central oxygen supply

c.       Wall suction outlet in treatment room

d.       Ventilator in treatment room

e.       Anesthetic gas scavenging in treatment room

f.        Dedicated recovery room

g.       Advanced pediatric airway equipment

h.       Defibrillator with pediatric pads/paddles

i.         Dedicated pediatric cardiopulmonary resuscitation cart

j.         Malignant hyperthermia cart

57. Which of the following personnel are on your anesthesia/sedation team during proton radiotherapy?

a.       Anesthesiologist

b.       Physician non-anesthesiologist

c.       Certified Nurse Anesthetist

d.       Anesthesia Assistant

e.       Anesthesiology Resident

f.        Registered Nurse/Nurses

g.       Anesthesia Technologist

h.       Other

58. Please specify which other personnel are on your anesthesia team during proton radiotherapy.

59. Is there a designated emergency medical response team within your proton radiotherapy facility?

a.       Yes

b.       No

60. Section Header: Anesthesia, Sedation, and Monitoring during Proton Radiotherapy

Do you routinely utilize non-pharmacologic methods to alleviate child anxiety before induction of anesthesia?

a.       Yes

b.       No

61. Which of the following non-pharmacologic methods do you routinely utilize to alleviate child anxiety before the start of proton radiotherapy? Please select all that may apply.

a.       Allowing family members into the treatment area

b.       The use of iPads/tablets/videos

c.       Child life/social education specialists

d.       Other

62. Please specify other methods of alleviating child anxiety prior to the start of proton radiotherapy.

63. What is your preferred method of anesthesia/sedation for children undergoing proton radiotherapy in the supine position?

a.       Total intravenous anesthesia without intubation

b.       Total intravenous anesthesia with Laryngeal Mask Airway

c.       Total intravenous anesthesia with endotracheal intubation

d.       Volatile anesthetics with endotracheal intubation

e.       Volatile anesthetics with Laryngeal Mask Airway

f.        Other

64. Please specify other preferred method of anesthesia for proton radiotherapy in the supine position.

65. Which of the following agents do you routinely administer during proton radiotherapy in the supine position? Please select all that may apply.

a.       Propofol

b.       Dexmedetomidine

c.       Opioids

d.       Anticholinergics

e.       IV Midazolam

f.        Oral midazolam

g.       Ketamine

h.       Barbiturates

i.         Halothane

j.         Sevoflurane

k.       Isoflurane

l.         Desflurane

m.     Antiemetics

n.       Other

66. Please specify other preferred drug

67. Do you administer anesthesia/sedation for proton radiotherapy in the prone position?

a.       Yes

b.       No

68. What is your preferred method of anesthesia/sedation for children undergoing proton radiotherapy in the prone position?

a.       Total intravenous anesthesia without intubation

b.       Total intravenous anesthesia with Laryngeal Mask Airway

c.       Total intravenous anesthesia with endotracheal intubation

d.       Volatile anesthetics with endotracheal intubation

e.       Volatile anesthetics with Laryngeal Mask Airway

f.        Other

69. Please specify other preferred method of anesthesia for proton radiotherapy in the prone position.

70. Which of the following agents do you routinely administer during proton radiotherapy in the prone position? Please select all that may apply.

a.       Propofol

b.       Dexmedetomidine

c.       Opioids

d.       Anticholinergics

e.       IV Midazolam

f.        Oral midazolam

g.       Ketamine

h.       Barbiturates

i.         Halothane

j.         Sevoflurane

k.       Isoflurane

l.         Desflurane

m.     Antiemetics

n.       Other

71. Please specify other preferred drug

72. Section Header: Post Anesthesia/Sedation Care

Approximately how many minutes does it take to transport a child from the treatment gantry to the dedicated recovery room after proton radiotherapy?

73. Which of the following are available at the dedicated recovery room after proton radiotherapy under anesthesia/sedation?

a.       Dedicated recovery room nurse

b.       Central oxygen supply

c.       Wall suction

d.       Monitors

74. Section Header: Monitoring Which of the following monitors do you use? Please select all that may apply.

During proton radiotherapy

a.       Pulse Oximetry

b.       ECG

c.       Blood Pressure

d.       Capnography

e.       Temperature

f.        None

75. During transport to the recovery room after proton radiotherapy

a.       Pulse Oximetry

b.       ECG

c.       Blood Pressure

d.       Capnography

e.       Temperature

f.        None

76 In the recovery room after proton radiotherapy checkbox

a.       Pulse Oximetry

b.       ECG

c.       Blood Pressure

d.       Capnography

e.       Temperature

f.        None

77. During proton radiotherapy, are you able to control the functions of your monitor from outside the treatment gantry?

a.       Yes

b.       No

78. In which year was your proton radiotherapy center opened?

79. Section Header: Experiences at your proton radiotherapy facility

Was your department consulted during the planning or construction phase of your proton therapy center?

a.       Yes

b.       No

80. Does the design or layout of your proton therapy center limit your anesthetic options?

a.       Yes

b.       No

81. Please list up to five building design or layout issues which negatively impact your practice at your proton radiotherapy location.

82 Section Header: Additional Comments or Explanations

Kindly provide any additional comments or explanations in the space provided.

83. Section Header: Form Status Complete?

a.       Incomplete

b.       Unverified

c.       Complete

Appendix B: introductory email

Re: Survey of sedation and non-sedation practices for children undergoing repetitive cranial or craniospinal radiotherapy

My name is Pascal Owusu-Agyemang. I am a pediatric anesthesiologist at the University of Texas MD Anderson Cancer Center. I hope this email finds you well.

I have received IRB approval to conduct a survey of sedation and non-sedation practices for children undergoing repetitive cranial or craniospinal radiotherapy. I would kindly like to request your participation, or help in identifying an anesthesiologist and/or a child-life specialist who may be willing to complete the survey. The survey is composed of questions about your preferred methods of facilitating radiotherapy with and/or without sedation, about your drug preferences, methods of monitoring, and recovery of patients after sedation. This survey will take about 15 minutes to complete, and you will be able to save and return to it for completion as often as needed. I will send the survey link to a willing participant.

Thank you very much for your consideration.

Sincerely,

Dr. Pascal Owusu-Agyemang

Appendix C: email to potential survey participants

Dear Colleague,

Extensive planning goes into the establishment of a radiotherapy center where children are treated. While there is some variability in sedation and non-sedation methods of facilitating treatment in children, there are also many common requirements and concerns. Much can be learned from the current practice of existing centers.

The primary objective of the following study is to collect information about anesthesia, sedation and non-sedation practices that are used to facilitate repetitive cranial or craniospinal radiotherapy in children. If you agree to take part in this survey, you will complete questions about methods of facilitating radiotherapy with and/or without sedation in children, about your drug preferences, methods of monitoring, and recovery of patients after sedation. This survey will take about 15 minutes to complete.

In accordance with the requirements of the institutional review board of the University of Texas MD Anderson Cancer Center, we require that you read the following consent statement before proceeding to the survey.

Consent Statement:

You have read the description of the study and have decided to participate in the research project described here. Survey responses will be collected anonymously, meaning that no HIPPA identifiers can be associated with the data, including name, institution (location smaller than a state), email address, IP address, etc. You understand that you may refuse to answer any (or all) of the questions at this or any other time. You understand that there is a possibility that you might be contacted in the future about this, but that you are free to refuse any further participation if you wish.

Please do not hesitate to contact the study chair Dr. Pascal Owusu-Agyemang, at 713-563-1646 or by email () if you have any further questions.

If you agree to participate, you may open the survey in your web browser by clicking the link below:

< https://redcap.mdanderson.org/surveys/?s=DDTW7ECF7W>

If the link above does not work, try copying the link below into your web browser.

This link is unique to you and should not be forwarded to others. After you have started the survey, you may save your responses as often as you would like and return to it for completion at a more convenient time.

Thank you for your interest in this survey.

Sincerely,

Drs. January Tsai, Ravish Kapoor, Acsa Zavala, and Pascal Owusu-Agyemang
